# Profiles and transitions of psychological adaptation in AI-assisted Japanese language learning

**DOI:** 10.3389/fpsyg.2026.1837484

**Published:** 2026-09-04

**Authors:** Yuanyuan Wu

**Affiliations:** School of Foreign Languages, Bengbu University, Bengbu, China

**Keywords:** AI-assisted Japanese language learning, latent profile transition analysis, learning burnout, psychological adaptation, resilience, technostress

## Abstract

As artificial intelligence becomes increasingly integrated into language learning, learners may differ in how they adapt psychologically to AI-assisted study. This study examined profiles and transitions of psychological adaptation to AI-assisted Japanese language learning and their associations with learning self-efficacy and burnout. A three-wave longitudinal survey was conducted across one academic semester, with 457 learners at Time 1. Technostress and psychological resilience served as indicators in latent profile and latent profile transition analyses. Three profiles were consistently identified: maladaptive adaptation, moderate adaptation, and positive adaptation. The maladaptive adaptation profile decreased from 26.48% at Time 1 to 17.74% at Time 3, whereas the positive adaptation profile increased from 27.35% to 35.73%. The positive adaptation profile showed the greatest temporal stability, while learners in the maladaptive adaptation profile most commonly transitioned to moderate adaptation. At Time 3, learners in the positive adaptation profile reported the highest Japanese learning self-efficacy and the lowest learning burnout, whereas those in the maladaptive adaptation profile showed the reverse pattern. By integrating the Job Demands–Resources framework with a person-centered longitudinal approach, this study demonstrates that psychological adaptation to AI-assisted language learning is heterogeneous, dynamic, and meaningfully associated with learners' confidence and burnout.

## 1 Introduction

As artificial intelligence becomes increasingly embedded in higher education, students are expected not only to use new digital tools but also to adapt psychologically to a more complex and demanding learning environment (Crompton and Burke, 2023). This issue is particularly salient in language learning, where AI-assisted platforms now provide automated feedback, personalized practice, speech recognition, and dialogue-based interaction that can reshape how learners engage with linguistic input and output. Recent studies have shown that AI-mediated language instruction can enhance learning achievement, self-regulated learning, and speaking performance, suggesting that AI has considerable pedagogical potential in second and foreign language education (Qiao and Zhao, 2023; Wei, 2023). Yet these benefits do not eliminate the need to understand how learners psychologically respond to such environments. From a psychological perspective, AI-assisted language learning is not simply an instructional innovation. It is also an adaptive challenge. Learners may experience greater autonomy, more immediate feedback, and more individualized support, but they may also encounter uncertainty about the reliability of AI-generated responses, cognitive overload caused by abundant information, and difficulty regulating their dependence on intelligent tools. Recent work has further suggested that AI-supported learning may influence engagement, procrastination, and other forms of academic functioning, indicating that the integration of AI into language education has consequences that extend well beyond technical efficiency (Zhai and Li, 2025). In this sense, the central issue is not merely whether AI can support language learning, but how learners psychologically manage the opportunities and pressures that accompany it.

A useful lens for understanding this process is psychological adaptation. In digitally intensive learning environments, one important source of maladjustment is technostress, which refers to strain arising from the demands of technology use. Recent research has shown that technostress remains relevant in student populations and is associated with time spent online, reduced quality of online learning, and less favorable educational experiences (Cazan et al., 2024; Saleem et al., 2024). At the same time, learners differ in the personal resources they bring to such environments. Research on college students suggests that resilience and self-efficacy may play protective roles when stress accumulates, buffering the negative effects of learning pressure and reducing the likelihood of academic burnout (Gong et al., 2023; Liu et al., 2024). These findings imply that learners' responses to AI-assisted language learning are likely to be shaped by the balance between technological strain and adaptive psychological resources. This perspective is especially important for learners of Japanese. AI-assisted Japanese language learning may offer substantial support for vocabulary practice, writing assistance, translation, and oral rehearsal, but it also requires learners to cope with a learning context marked by high informational density, ongoing self-monitoring, and the need to judge the appropriateness of AI-generated output. Under such conditions, learners are unlikely to respond in identical ways. Some may experience AI as motivating and manageable, whereas others may find it stressful or psychologically destabilizing. Moreover, these patterns of adaptation may not remain fixed, but may evolve over time as learners accumulate experience and become more or less comfortable with AI-assisted study.

Against this background, the present study investigates psychological adaptation to AI-assisted Japanese language learning from a person-centered and longitudinal perspective. Rather than assuming that all learners respond similarly to AI-assisted study, the study examines whether distinct profiles can be identified on the basis of technostress and resilience, whether these profiles remain stable or change across time, and whether they differ in Japanese learning self-efficacy and learning burnout. By doing so, the study aims to position AI-assisted Japanese language learning as a genuinely psychological topic, one that concerns not only technological adoption but also learners' stress, coping, confidence, and wellbeing.

## 2 Literature review and research questions

### 2.1 AI-Assisted Japanese language learning as a context of psychological adaptation

Artificial intelligence has become increasingly visible in language education, where it is used to support tutoring, feedback, dialogue practice, translation, automated evaluation, and self-directed learning. Recent reviews suggest that AI-assisted language learning is no longer a marginal innovation but an expanding instructional ecology that is reshaping how learners access language input, practice output, and regulate their own learning processes (Alshumaimeri and Alshememry, 2024; Jiang, 2022; Li et al., 2025; Zhai and Wibowo, 2023). Although much of the empirical evidence has been generated in English language education, the broader implication is clear: AI technologies are changing the psychological conditions under which language learning takes place. The growing body of quantitative evidence also points to the educational potential of AI in language learning. Meta-analytic findings indicate that AI-assisted language learning can improve language achievement and support learners' self-regulation, particularly in technology-mediated and out-of-class contexts (Guan et al., 2024; Lee and Lee, 2022; Xu and Wang, 2024). At the same time, recent studies on ChatGPT and related tools suggest that learners often perceive AI as flexible, accessible, and useful for self-directed language practice, revision, and feedback (Li et al., 2024; Teng, 2024). These benefits are especially relevant to language learning, where repeated practice, individualized feedback, and low-stakes interaction are central to progress. However, AI-assisted language learning is not simply a more efficient version of traditional learning. It also introduces new demands that can affect learners psychologically. Students are now expected to judge the quality of AI-generated responses, formulate effective prompts, manage the temptation of overreliance, and navigate uncertain norms about appropriate use (Barrett and Pack, 2023; Li et al., 2024; Teng, 2024). In this sense, AI-assisted Japanese language learning should be understood not merely as a technological environment, but as a psychologically consequential learning context. This is particularly important for learners of Japanese, whose study often involves sustained attention to linguistic form, sociolinguistic appropriateness, and script-related complexity. The adaptive challenge, therefore, lies not only in using AI tools, but also in maintaining a healthy psychological balance while learning with them.

### 2.2 Technostress and resilience in AI-assisted language learning

A useful starting point for understanding this adaptive challenge is the concept of technostress. Early research conceptualized technostress as the strain individuals experience when they are unable to cope effectively with information and communication technologies (Ragu-Nathan et al., 2008; Tarafdar et al., 2007). Subsequent work has described technostress as a multidimensional construct involving overload, complexity, insecurity, uncertainty, and perceived invasion, all of which may undermine performance and wellbeing when technology becomes demanding rather than supportive (Kumar, 2024; Ragu-Nathan et al., 2008; Tarafdar et al., 2007). Although technostress was initially examined in organizational settings, more recent research has shown that it is also highly relevant in educational contexts, particularly among university students learning in technology-enhanced environments (Wang et al., 2021). In AI-assisted language learning, technostress may emerge in several ways. Learners may feel overloaded by the speed and volume of AI-generated information, uncertain about whether AI output is accurate, insecure about their own competence relative to intelligent systems, or cognitively burdened by the need to monitor and regulate their use of multiple tools. These experiences are not incidental. Rather, they represent a central part of the learner's adaptation to a digital learning environment. When technostress accumulates, it can erode confidence, reduce satisfaction, and interfere with productive engagement in learning tasks (Kumar, 2024; Wang et al., 2021).

Yet psychological adaptation in such a context cannot be understood solely in terms of strain. It is equally important to consider the personal resources that enable learners to respond constructively to difficulty. One such resource is psychological resilience, commonly defined as the capacity to adapt successfully to challenge, recover from setbacks, and continue functioning effectively under stress (Campbell-Sills and Stein, 2007; Connor and Davidson, 2003). In higher education research, resilience has been linked to academic persistence, better mental health, stronger wellbeing, and more adaptive responses to adversity (Etherton et al., 2022; Hartley, 2011). Rather than functioning as the simple absence of strain, resilience represents an active capacity to mobilize coping effort, preserve goal-directed behavior, and recover when technological demands disrupt learning.

To explain why technostress and resilience jointly define psychological adaptation, the present study draws on Job Demands–Resources (JD–R) theory and its extension to educational settings (Bakker and Demerouti, 2017; Demerouti et al., 2001; Jagodics and Szabó, 2023). The JD–R framework proposes that functioning reflects the balance between demands that consume psychological energy and resources that enable individuals to manage those demands and sustain engagement. In AI-assisted Japanese language learning, technostress represents a context-specific demand because overload, invasion, complexity, insecurity, and uncertainty require continued cognitive and emotional regulation. Resilience represents a personal resource because it supports recovery, flexible coping, and continued engagement when AI-generated feedback is difficult, ambiguous, or unreliable.

From this perspective, psychological adaptation is not adequately represented by either construct alone. Technostress indicates the magnitude of demands confronting the learner, whereas resilience indicates the capacity available to absorb and manage those demands. Their joint configuration therefore captures the learner's adaptive position within the AI-assisted environment: a demand-dominant configuration (high technostress and low resilience) reflects vulnerability to maladaptation; a resource-advantaged configuration (low technostress and high resilience) reflects positive adaptation; and intermediate configurations reflect a more tentative balance between demands and resources. This framework gives the proposed profiles substantive psychological meaning rather than treating technostress and resilience as merely correlated characteristics. Accordingly, the present study conceptualizes psychological adaptation as a person-specific demand–resource configuration that may remain stable or change as learners gain experience with AI-assisted Japanese language learning.

### 2.3 A person-centered perspective on psychological adaptation

Most prior research on technology-related learning processes has adopted a variable-centered approach. Such studies have been valuable in identifying associations among stress, motivation, self-efficacy, and learning outcomes, but they tend to focus on average relationships across the whole sample (Jiang, 2022; Wang et al., 2021). This strategy implicitly assumes that learners respond to AI-assisted learning in broadly similar ways. In reality, however, learners may differ markedly in how they combine technological strain with adaptive psychological resources.

A person-centered perspective is theoretically appropriate because demand–resource theory does not imply that technostress and resilience operate as isolated characteristics with uniform consequences. Instead, adaptation depends on how contextual demands and personal resources are configured within the same learner (Bakker and Demerouti, 2017; Demerouti et al., 2001; Jagodics and Szabó, 2023). The same level of technostress may have different psychological meaning when accompanied by strong rather than weak resilience, and resilience may provide limited protection when technological demands consistently exceed the learner's coping capacity. Consequently, averaging the independent associations of technostress and resilience can conceal distinct adaptive conditions that exist within the learner population. From this demand–resource perspective, several qualitatively different configurations are theoretically meaningful. A demand-dominant configuration, in which technostress is high and resilience is low, indicates that the learner's coping resources are insufficient relative to the demands of AI-assisted learning and therefore represents vulnerability to maladaptation. An intermediate configuration, in which neither demands nor resources clearly predominate, represents a more provisional and potentially transitional adaptive state. A resource-advantaged configuration, characterized by lower technostress and higher resilience, indicates that the learner possesses sufficient capacity to regulate technological demands and sustain adaptive engagement. These configurations differ not merely in overall score level but in the psychological relation between experienced demands and available coping resources. Latent profile analysis is therefore used as an empirical means of identifying theoretically meaningful demand–resource configurations across continuous indicators (Ferguson et al., 2020; Nylund et al., 2007).

This perspective becomes even more informative when psychological adaptation is examined longitudinally. Adaptation to AI-assisted learning is unlikely to be static, especially across an academic semester during which learners accumulate experience, form routines, and revise their beliefs about AI use. Latent profile transition analysis extends the person-centered approach by examining whether and how individuals move between profiles over time (Nylund-Gibson et al., 2023; Ryoo et al., 2018). This allows researchers to distinguish relatively stable patterns from more dynamic ones and to determine whether learners tend to remain in maladaptive states, shift toward more positive adaptation, or fluctuate between profiles as their experience evolves. Accordingly, a person-centered longitudinal approach is particularly well suited to the present study. It acknowledges that AI-assisted Japanese language learning may evoke different combinations of stress and resilience across learners, and it further recognizes that these combinations may change rather than remain fixed.

### 2.4 Psychological adaptation profiles and distal learning outcomes

If latent adaptation profiles are substantively meaningful, they should also be associated with important learning-related outcomes. In the present study, two such outcomes are especially relevant: Japanese learning self-efficacy and learning burnout.

Self-efficacy and learning burnout can be understood as complementary outcomes of the same demand–resource configuration rather than as unrelated distal variables. Self-efficacy refers to learners' beliefs about their capability to perform domain-specific tasks successfully. Social cognitive theory proposes that such beliefs are strengthened when learners can exercise control, persist through difficulty, and interpret successful performance as evidence of mastery (Bandura, 1997; Fryer et al., 2020; Wang et al., 2014). In AI-assisted Japanese language learning, technostress can disrupt these processes by directing attention toward tool complexity, output uncertainty, and the continuing need to monitor AI-generated responses, thereby weakening perceived control over learning. Resilience supports the complementary process by helping learners recover from errors, adjust strategies, and persist when AI feedback is imperfect. Lower technostress and higher resilience should therefore jointly create a resource-advantaged condition in which successful AI-supported practice is more likely to be experienced as controllable mastery, supporting stronger Japanese learning self-efficacy.

Learning burnout, characterized by exhaustion, cynicism, and reduced academic efficacy, represents the strain-related side of the same adaptive process (Hu and Schaufeli, 2009; Schaufeli et al., 2002). Job Demands–Resources theory distinguishes a health-impairment process, through which sustained demands consume psychological energy, from a resource-supported process that preserves coping and engagement (Bakker and Demerouti, 2017; Demerouti et al., 2001; Jagodics and Szabó, 2023). In AI-assisted Japanese language learning, technological overload, complexity, and uncertainty require repeated cognitive and emotional effort. When elevated technostress is coupled with low resilience, learners face a resource-deficit condition that restricts recovery and increases the accumulation of exhaustion and cynical detachment. When technostress is lower and resilience is stronger, technological demands consume fewer psychological resources, while the capacity to recover and remain engaged is greater, reducing vulnerability to learning burnout.

Accordingly, self-efficacy and learning burnout are treated as complementary distal outcomes of demand–resource adaptation rather than as unrelated variables. The demand–resource perspective provides a theoretical basis for expecting the empirically derived profiles to differ meaningfully in both outcomes. However, because the number and configuration of the profiles are not specified in advance, the precise pattern and magnitude of these differences are examined through research questions rather than directional hypotheses. This framing establishes self-efficacy and learning burnout as external manifestations through which the substantive meaning of the identified adaptation profiles can be evaluated.

### 2.5 The present study

The present study addresses several gaps in the emerging literature on AI-assisted language learning. First, although AI has been widely discussed in language education, existing research has focused predominantly on learning effectiveness, perceptions of specific tools, or single psychological variables, with much less attention to learners' broader patterns of psychological adaptation (Jiang, 2022; Li et al., 2025; Xu and Wang, 2024). Second, prior studies have generally relied on variable-centered approaches, which are less suited to identifying heterogeneity in how learners respond to AI-assisted learning environments. Third, longitudinal evidence remains limited, despite the fact that adaptation to AI use is likely to evolve over time. Finally, relatively little work has linked latent adaptation patterns to domain-relevant outcomes such as language learning self-efficacy and learning burnout. To address these gaps, the present study examines psychological adaptation to AI-assisted Japanese language learning from a person-centered and longitudinal perspective. Specifically, it uses technostress and resilience as core indicators of adaptation, identifies latent profiles across three waves, examines transitions between these profiles over time, and investigates whether profile membership is associated with Japanese learning self-efficacy and learning burnout.

Based on the foregoing theoretical framework and gaps in the literature, the present study addressed the following research questions.

**RQ1:** what distinct latent profiles of psychological adaptation can be identified among learners of Japanese based on technostress and resilience?

**RQ2:** what patterns of stability and transition occur among the identified psychological adaptation profiles across the three waves?

**RQ3:** how does Japanese learning self-efficacy differ across the psychological adaptation profiles identified at Time 3?

**RQ4:** how does learning burnout differ across the psychological adaptation profiles identified at Time 3?

## 3 Methods

### 3.1 Research design and participants

The present study employed a three-wave longitudinal survey design to investigate profiles and transitions of psychological adaptation to AI-assisted Japanese language learning among learners of Japanese. Data were collected over the course of one academic semester. The first wave was administered in September 2025, the second wave in November 2025, and the third wave in January 2026, with an interval of approximately 8 weeks between adjacent waves. This design was intended to capture both short-term stability and meaningful change in learners' psychological responses as AI-assisted Japanese language learning became progressively integrated into their regular study routines. Participants were recruited from Japanese language courses offered at public universities in mainland China. To be eligible for the study, participants had to meet two criteria. First, they had to be currently enrolled in a Japanese language course at the tertiary level. Second, they had to have used at least one AI-based tool for Japanese learning during the current semester, such as an AI ChatBot, an AI writing assistant, an AI translation tool, or an AI-supported speaking practice application.

Before the formal survey, a pilot study was conducted with 32 learners of Japanese to assess the clarity and contextual appropriateness of the adapted questionnaire. Based on participant feedback, minor wording revisions were made to several items concerning technostress and language learning self-efficacy. The pilot responses were not included in the main analyses. At Time 1, 486 questionnaires were returned. After excluding 29 responses because of duplicate submissions, patterned responding, or completion times shorter than one third of the median response time, 457 valid responses were retained. Of these participants, 421 completed the Time 2 survey and 389 completed the Time 3 survey. A total of 366 respondents completed all three waves, yielding a panel retention rate of 80.09% relative to the valid Time 1 sample. Because missing data were handled with full information maximum likelihood estimation, all 457 participants were retained in the longitudinal analyses (Enders and Bandalos, 2001). The analytic sample consisted of learners drawn from different year levels and from both major and non-major Japanese courses. Detailed demographic characteristics of the sample are reported in the Results section.

### 3.2 Procedure

All questionnaires were administered online in Chinese. The survey link was remained open for 7 days at each wave. To reduce common method inflation, the focal profile indicators were collected repeatedly across three time points, and the distal outcome variables were assessed only at the third wave. In addition, participants were informed that there were no right or wrong answers and that their responses would be used for research purposes only. The questionnaire included four sections. The first section collected demographic and background information, including age, gender, year of study, major status, self-rated Japanese proficiency, frequency of AI use for Japanese learning, and prior experience with formal AI-related training. The second section assessed technostress in AI-assisted Japanese learning. The third section assessed psychological resilience. The fourth section, administered only at Time 3, measured Japanese learning self-efficacy and learning burnout as distal outcomes associated with the latent adaptation profiles.

### 3.3 Measures

#### 3.3.1 Technostress in AI-Assisted Japanese language learning

Technostress was assessed at each wave using an adapted version of the technostress creators instrument developed for university students in technology-enhanced learning by Wang et al. (2021). The original scale consists of 22 items covering five dimensions, namely techno-overload, techno-invasion, techno-complexity, techno-insecurity, and techno-uncertainty. In the present study, the wording of the items was minimally adapted so that the target context referred specifically to AI-assisted Japanese language learning rather than technology-enhanced learning in general. For instance, references to digital learning tools were replaced by references to AI tools used for vocabulary learning, writing support, reading comprehension, translation, and speaking practice. The adaptation process followed translation and cross-context adjustment procedures recommended in previous scale adaptation research (Sousa and Rojjanasrirat, 2011). All items were rated on a five-point Likert scale ranging from 1 for strongly disagree to 5 for strongly agree. Mean scores were computed for each of the five dimensions, with higher scores indicating stronger perceived technostress.

#### 3.3.2 Psychological resilience

Psychological resilience was measured at each wave using the 10-item Connor–Davidson Resilience Scale short form (Campbell-Sills and Stein, 2007), which was derived from the original Connor–Davidson Resilience Scale (Connor and Davidson, 2003). The Chinese version validated by Wang et al. (2010) was used in the present study. This instrument assesses an individual's capacity to recover from setbacks, remain effective under pressure, and adapt to changing demands. Items were rated on a five-point scale ranging from 0 for not true at all to 4 for true nearly all the time. Following previous research, a mean score was computed across the 10 items, with higher scores indicating greater resilience.

#### 3.3.3 Japanese learning self-efficacy

Japanese learning self-efficacy was measured at Time 3 using an adapted version of the Questionnaire of English Self-Efficacy validated by Wang et al. (2014). The original instrument contains 32 items assessing self-efficacy in four language domains, namely listening, speaking, reading, and writing. In the present study, the wording was adapted by replacing English with Japanese while preserving the original task structure and response format. Because self-efficacy is inherently domain specific, this language-specific measure was considered more appropriate than a general academic self-efficacy instrument. Items were rated on a seven-point scale ranging from 1 for cannot do at all to 7 for highly certain can do. A mean score across all items was used as the overall indicator of Japanese learning self-efficacy, with higher scores reflecting stronger confidence in performing Japanese language tasks.

#### 3.3.4 Learning burnout

Learning burnout was measured at Time 3 using the Maslach Burnout Inventory–Student Survey (Schaufeli et al., 2002). To ensure suitability for Chinese learners, the present study followed the Chinese validation study conducted by Hu and Schaufeli (2009). The instrument contains 15 items covering three components of student burnout, namely exhaustion, cynicism, and reduced academic efficacy. Responses were recorded on a seven-point frequency scale ranging from 0 for never to 6 for always. Following established practice, the academic efficacy items were reverse coded so that higher scores consistently reflected higher burnout. A mean score was then calculated across all items.

#### 3.3.5 Control variables

Seven background variables were included as control variables: gender, age, year of study, major status, self-rated Japanese proficiency, frequency of AI use for Japanese learning, and prior AI-related training. Frequency of AI use was assessed with a single item ranging from 1 (less than once a week) to 5 (almost every day). Gender was coded 0 for male and 1 for female, major status was coded 0 for non-major and 1 for Japanese major, and prior AI-related training was coded 0 for no and 1 for yes. In the auxiliary analyses, all seven variables were entered simultaneously as predictors of Time 1 profile membership and as covariates in the adjusted Time 3 distal-outcome models. They were not specified as predictors of the longitudinal transition probabilities.

### 3.4 Data analysis

Analyses used R version 4.3.2 and Mplus version 8.10. Descriptive and measurement analyses used tidyverse version 2.0.0 and lavaan version 0.6-17, respectively (Rosseel, 2012), while mixture models were run through MplusAutomation version 1.1.0 (Hallquist and Wiley, 2018).

Data analysis proceeded in five stages.

#### 3.4.1 First, preliminary and measurement analyses

Descriptive statistics, correlations, reliability coefficients, confirmatory factor analyses, and longitudinal measurement invariance tests were conducted. Measurement models were evaluated using CFI, TLI, RMSEA, and SRMR (Vandenberg and Lance, 2000; Hu and Bentler, 1999; Chen, 2007).

#### 3.4.2 Second, latent profile analyses

One- through six-profile models were estimated at each wave using robust maximum likelihood and compared using information criteria, entropy, class size, interpretability, LMR-LRT, and BLRT (Nylund et al., 2007). Models used STARTS = 500 100, STITERATIONS = 500, 1,000 maximum final iterations, a **10**^**−6**^ convergence criterion, and STSEED = 202509.

#### 3.4.3 Third, latent profile transition analysis

The three-wave model was selected by comparing increasingly constrained measurement models (Nylund-Gibson et al., 2023; Ryoo et al., 2018). Missing data were handled using full information maximum likelihood, allowing all 457 participants to contribute (Enders and Bandalos, 2001).

#### 3.4.4 Fourth, distal-outcome analysis

Time 3 profile differences in self-efficacy and burnout were tested using BCH-adjusted Wald tests and pairwise comparisons, thereby accounting for classification uncertainty (Asparouhov and Muthén, 2014; Bolck et al., 2004).

#### 3.4.5 Fifth, auxiliary analyses

The seven control variables predicted Time 1 profile membership using R3STEP but were not used to predict transitions. BCH-weighted regressions tested whether adjusted Time 3 outcome means differed across profiles (Asparouhov and Muthén, 2014; Bolck et al., 2004).

## 4 Results

### 4.1 Sample characteristics and preliminary analyses

A total of 457 valid responses were retained at Time 1 and entered into the main analyses. Among these participants, 421 completed the Time 2 survey and 389 completed the Time 3 survey, with 366 learners providing complete data across all three waves. The demographic characteristics of the Time 1 sample are presented in Table 1. The sample was predominantly female (76.1%), and the distribution across year levels was relatively balanced. More than half of the participants were enrolled in non-major Japanese courses (56.0%), and intermediate learners represented the largest proficiency group (45.7%). Most participants reported no prior AI-related training (62.1%), whereas the most common frequency of AI use was 3–4 times a week (30.0%). Descriptive statistics and internal consistency coefficients for all study variables are reported in Table 2. The internal consistency of the multi-item measures was acceptable to good across the three waves, with Cronbach's alpha coefficients ranging from 0.764 to 0.930. In terms of mean-level change, all five technostress dimensions showed a gradual decline from Time 1 to Time 3. By contrast, psychological resilience increased modestly across the three measurement occasions, rising from 2.03 at Time 1 to 2.19 at Time 3. At Time 3, the mean level of Japanese learning self-efficacy was 4.67, whereas the mean level of learning burnout was 2.55.

**Table 1:** Demographic characteristics of the Time 1 sample.

Variable	Category	*n*	%
Gender	Female	348	76.15
	Male	109	23.85
Year of study	First year	132	28.88
	Second year	121	26.48
	Third year	110	24.07
	Fourth year	94	20.57
Course type	Japanese major	201	43.98
	Non-major Japanese course	256	56.02
Self-rated proficiency	Beginning	125	27.35
	Intermediate	209	45.73
	Advanced	123	26.91
Prior AI-related training	Yes	173	37.86
	No	284	62.14
Frequency of AI use	Less than once a week	61	13.35
	1–2 times a week	104	22.76
	3–4 times a week	137	29.98
	5–6 times a week	94	20.57
	Almost every day	61	13.35

*N*, 457.

Percentages may not total 100.00 because of rounding.

**Table 2:** Descriptive statistics and internal consistency of the study variables.

Time	Variable	*M*	*SD*	Cronbach's α
T1	Techno-overload	3.13	0.80	0.768
	Techno-invasion	3.02	0.84	0.790
	Techno-complexity	3.08	0.82	0.782
	Techno-insecurity	2.96	0.83	0.793
	Techno-uncertainty	2.96	0.85	0.799
	Psychological resilience	2.03	0.63	0.835
T2	Techno-overload	3.04	0.82	0.765
	Techno-invasion	2.85	0.86	0.817
	Techno-complexity	2.97	0.82	0.788
	Techno-insecurity	2.79	0.84	0.779
	Techno-uncertainty	2.84	0.85	0.806
	Psychological resilience	2.12	0.63	0.843
T3	Techno-overload	2.84	0.83	0.783
	Techno-invasion	2.83	0.79	0.764
	Techno-complexity	2.81	0.83	0.784
	Techno-insecurity	2.66	0.84	0.797
	Techno-uncertainty	2.78	0.87	0.805
	Psychological resilience	2.19	0.65	0.839
	Japanese learning self-efficacy	4.67	0.96	0.925
	Learning burnout	2.55	1.05	0.930

Descriptive statistics are based on the available data at each wave.

Table 3 presents the correlations among the composite study variables. Technostress was consistently and negatively associated with psychological resilience both within and across waves. It was also negatively related to Japanese learning self-efficacy and positively related to learning burnout. Psychological resilience showed the opposite pattern, being positively associated with Japanese learning self-efficacy and negatively associated with learning burnout. In addition, the same constructs demonstrated moderate temporal stability across the three waves. Overall, these preliminary results provide initial support for the expected relationships among technostress, resilience, and the two distal learning outcomes.

**Table 3:** Correlations among the composite study variables.

Variable	1	2	3	4	5	6	7	8
1. Technostress T1	—	−0.80	0.56	−0.44	0.41	−0.35	−0.19	0.24
2. Resilience T1	−0.80	—	−0.47	0.39	−0.35	0.35	0.17	−0.20
3. Technostress T2	0.56	−0.47	—	−0.72	0.56	−0.48	−0.27	0.36
4. Resilience T2	−0.44	0.39	−0.72	—	−0.49	0.42	0.24	−0.39
5. Technostress T3	0.41	−0.35	0.56	−0.49	—	−0.77	−0.41	0.60
6. Resilience T3	−0.35	0.35	−0.48	0.42	−0.77	—	0.41	−0.51
7. Japanese learning self-efficacy T3	−0.19	0.17	−0.27	0.24	−0.41	0.41	—	−0.24
8. Learning burnout T3	0.24	−0.20	0.36	−0.39	0.60	−0.51	−0.24	—

Technostress at each wave was represented by the composite score of the five technostress dimensions for the correlation analysis.

All correlation coefficients were statistically significant at *p* < 0.05.

### 4.2 Measurement models and longitudinal measurement invariance

Confirmatory factor analyses were first conducted to examine the factorial validity of the technostress and resilience measures at each wave. The hypothesized measurement models showed acceptable fit across the three time points, indicating that the latent structure of the focal constructs was adequately represented in the present sample. On this basis, longitudinal measurement invariance was further tested to determine whether the profile indicators could be meaningfully compared over time. As reported in Table 4, the configural invariance models for both technostress and resilience demonstrated acceptable fit, suggesting that the same basic factor structure was retained across the three waves. For technostress, the metric invariance model showed only a very small decline in fit relative to the configural model (ΔCFI = −0.002, ΔRMSEA = 0.001), and the scalar invariance model likewise remained within recommended thresholds (ΔCFI = −0.005, ΔRMSEA = 0.001). A similar pattern was observed for resilience. Compared with the configural model, the metric invariance model showed only minimal changes in fit (ΔCFI = −0.004, ΔRMSEA = 0.002), and the scalar invariance model also remained acceptable (ΔCFI = −0.009, ΔRMSEA = 0.005). These results support the longitudinal comparability of the technostress and resilience measures across the three waves. This provides an adequate measurement foundation for the subsequent latent profile and latent profile transition analyses.

**Table 4:** Fit indices for longitudinal measurement invariance models.

Construct	Model	*X* ^2^	*df*	CFI	TLI	RMSEA	SRMR	ΔCFI	ΔRMSEA
Technostress	Configural invariance	1,085.45	510	0.972	0.968	0.046	0.041	—	—
	Metric invariance	1,142.20	548	0.970	0.967	0.047	0.044	−0.002	0.001
	Scalar invariance	1,228.35	586	0.965	0.962	0.048	0.046	−0.005	0.001
	Configural invariance	235.12	105	0.975	0.969	0.048	0.035	—	—
	Metric invariance	285.40	123	0.971	0.967	0.050	0.042	−0.004	0.002
	Scalar invariance	378.15	141	0.962	0.958	0.055	0.049	−0.009	0.005

*N*, 457.

Change indices are calculated relative to the immediately preceding model.

### 4.3 Latent profile analyses

Latent profile analyses were conducted separately at each wave using the five technostress dimensions and psychological resilience as profile indicators. Models with one to six profiles were estimated, and the fit statistics are presented in Table 5. Across all three waves, the three-profile solution showed the most favorable overall pattern of fit. At Time 1, the three-profile model yielded lower AIC, BIC, and adjusted BIC values than the one- and two-profile models, together with high classification accuracy (entropy = 0.906) and significant LMR and BLRT results. A similar pattern emerged at Time 2 and Time 3, where the three-profile solution again showed the lowest information criteria among the substantively plausible models, high entropy values of 0.879 and 0.929, and significant likelihood ratio tests. Although additional profiles were estimated, these more complex solutions did not yield consistent improvements in fit and in some cases resulted in relatively small classes. Taken together, the three-profile solution was retained at each wave because it provided the best balance of statistical fit, classification precision, parsimony, and substantive interpretability.

**Table 5:** Fit indices for latent profile models at each wave.

Wave	Profiles	AIC	BIC	aBIC	Entropy	LMR *p*	BLRT *p*	Smallest class (%)
T1	1-profile	3910.74	4022.10	3936.41	—	—	—	—
	2-profile	3896.33	4040.69	3929.61	0.640	< 0.001	0.010	47.92
	3-profile	**3,815.22**	**3,992.58**	**3,856.11**	**0.906**	< 0.001	**0.005**	**26.91**
	4-profile	3,824.49	4,034.84	3,872.99	0.779	0.566	0.840	14.88
	5-profile	3,824.57	4,067.93	3,880.68	0.789	0.150	0.385	7.66
	6-profile	3,826.12	4,102.47	3,889.84	0.794	0.150	0.430	8.10
	1-profile	3,725.65	3,834.80	3,749.12	—	—	—	—
	2-profile	3,709.36	3,850.85	3,739.78	0.658	< 0.001	0.010	47.03
	**3-profile**	**3,650.88**	**3,824.71**	**3,688.26**	**0.879**	< 0.001	**0.005**	**19.48**
	4-profile	3,656.88	3,859.34	3,697.50	0.784	0.150	0.395	16.15
	5-profile	3,658.88	3,890.75	3,703.53	0.816	0.150	0.350	3.56
	6-profile	3,660.88	3,926.03	3,713.42	0.835	0.150	0.550	1.90
	1-profile	3,455.11	3,562.12	3,476.46	—	—	—	—
	2-profile	3,438.00	3,576.72	3,465.67	0.712	< 0.001	0.005	37.53
	**3-profile**	**3,349.38**	**3,519.81**	**3,383.37**	**0.929**	< 0.001	**0.005**	**17.74**
	4-profile	3,361.45	3,563.59	3,401.77	0.793	0.863	0.960	11.83
	5-profile	3,363.84	3,597.69	3,410.49	0.808	0.150	0.565	4.63
	6-profile	3,364.27	3,629.83	3,417.24	0.783	0.150	0.400	3.86

AIC, Akaike information criterion; BIC, Bayesian information criterion; aBIC, sample-size adjusted Bayesian information criterion; LMR, Lo–Mendell–Rubin adjusted likelihood ratio test; BLRT, bootstrap likelihood ratio test.

The retained three-profile solution at each wave is highlighted in bold. Smallest-class percentages are model-estimated proportions and may differ slightly from percentages based on most likely profile assignment.

The retained profiles showed a highly similar structure across the three measurement occasions. The first profile was characterized by comparatively high levels of technostress across all five dimensions and low levels of resilience. This profile was therefore labeled *maladaptive adaptation*. The second profile showed values close to the sample mean on most indicators and was labeled *moderate adaptation*. The third profile displayed comparatively low levels of technostress and high levels of resilience and was labeled *positive adaptation*. The consistency of these profile patterns across the three waves supported the conceptual stability of the classification solution over time.

The proportions of learners assigned to each profile at each wave are reported in Table 6. At Time 1, 26.48% of learners were classified into the maladaptive adaptation profile, 46.17% into the moderate adaptation profile, and 27.35% into the positive adaptation profile. At Time 2, the proportion of learners in the maladaptive adaptation profile decreased to 19.71%, whereas the proportion in the positive adaptation profile increased to 33.73%. This pattern continued at Time 3, with the maladaptive adaptation profile further decreasing to 17.74% and the positive adaptation profile increasing to 35.73%. Across all three waves, the moderate adaptation profile remained the largest group, accounting for approximately 46% of the sample. Overall, these results suggest that psychological adaptation to AI-assisted Japanese language learning was heterogeneous in nature, with a gradual shift from less adaptive to more adaptive profiles over time.

**Table 6:** Profile membership across the three waves.

Profile	T1 *n* (%)	T2 *n* (%)	T3 *n* (%)
Maladaptive adaptation	121 (26.48)	83 (19.71)	69 (17.74)
Moderate adaptation	211 (46.17)	196 (46.56)	181 (46.53)
Positive adaptation	125 (27.35)	142 (33.73)	139 (35.73)

Percentages are based on the most likely estimated latent profile membership at each wave.

### 4.4 Latent profile transition analysis

**Figure 1:**
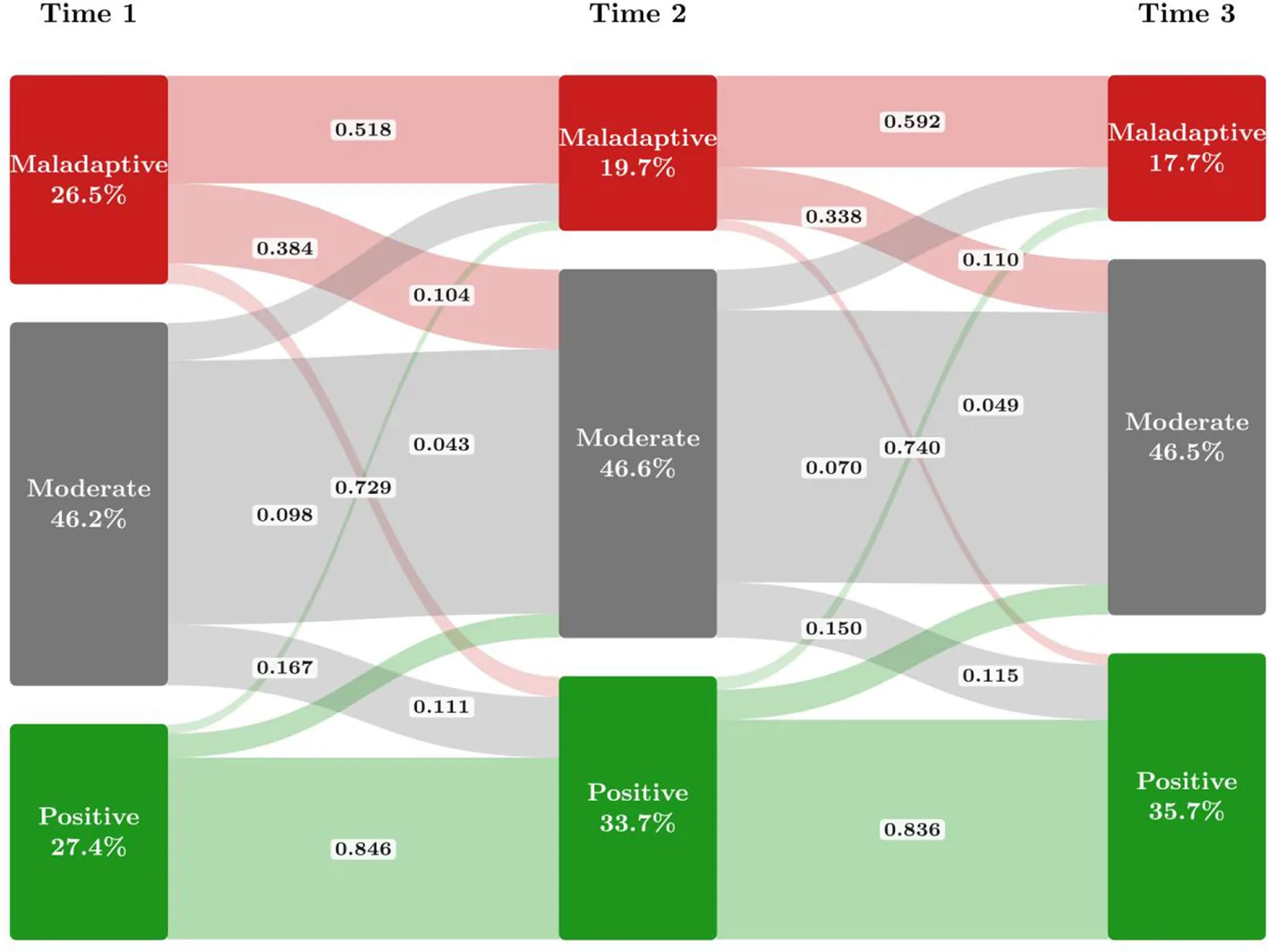
Three-wave latent profile transitions of psychological adaptation to AI-assisted Japanese language learning.

After establishing the three-profile solution at each wave, a three-wave latent profile transition analysis was conducted to examine the stability and change of profile membership over time. The transition probabilities are presented in Table 7, and the corresponding transition patterns are illustrated in Figure 1. Overall, the moderate adaptation profile constituted the largest group across all three measurement occasions, accounting for 46.17% of the sample at Time 1, 46.56% at Time 2, and 46.53% at Time 3. By contrast, the proportion of learners in the maladaptive adaptation profile decreased steadily over time, from 26.48% at Time 1 to 19.71% at Time 2 and 17.74% at Time 3. At the same time, the positive adaptation profile increased from 27.35% at Time 1 to 33.73% at Time 2 and 35.73% at Time 3. This pattern suggests a gradual shift toward more adaptive psychological responses to AI-assisted Japanese language learning across the semester. The transition probabilities further revealed meaningful differences in profile stability. Learners in the positive adaptation profile showed the highest temporal stability, with 84.6% remaining in the same profile from Time 1 to Time 2 and 83.6% remaining stable from Time 2 to Time 3. The moderate adaptation profile also demonstrated substantial continuity, with stability probabilities of 72.9% and 74.0% across the two transition intervals. In contrast, the maladaptive adaptation profile showed comparatively lower stability, with 51.8% of learners remaining in this profile from Time 1 to Time 2 and 59.2% from Time 2 to Time 3.

**Table 7:** Transition probabilities between latent profiles.

Initial profile	Time 1→Time 2			Time 2→Time 3		
	Maladaptive	Moderate	Positive	Maladaptive	Moderate	Positive
Maladaptive adaptation	0.518	0.384	0.098	0.592	0.338	0.070
Moderate adaptation	0.104	0.729	0.167	0.110	0.740	0.150
Positive adaptation	0.043	0.111	0.846	0.049	0.115	0.836

Rows represent profile membership at the earlier wave, and columns represent profile membership at the subsequent wave.

In addition to within-profile stability, the results also indicated meaningful cross-profile movement. Among learners initially classified in the maladaptive adaptation profile, the most common transition was into the moderate adaptation profile, with probabilities of 38.4% from Time 1 to Time 2 and 33.8% from Time 2 to Time 3. Direct transitions from maladaptive adaptation to positive adaptation were relatively uncommon, accounting for 9.8% and 7.0% across the two intervals, respectively. Learners in the moderate adaptation profile were more likely to move toward the positive adaptation profile than toward the maladaptive adaptation profile, with corresponding probabilities of 16.7% vs. 10.4% from Time 1 to Time 2 and 15.0% vs. 11.0% from Time 2 to Time 3. For learners in the positive adaptation profile, shifts into less adaptive patterns were infrequent, particularly transitions into the maladaptive adaptation profile, which remained below 5% across both intervals. These findings indicate that psychological adaptation to AI-assisted Japanese language learning was characterized by both continuity and change. Although most learners remained in the same profile over time, the observed shifts were not random. Instead, the transition pattern suggested a general movement away from maladaptive adaptation and toward more adaptive forms of psychological functioning over the course of the semester.

### 4.5 Distal outcomes of profile membership

**Figure 2:**
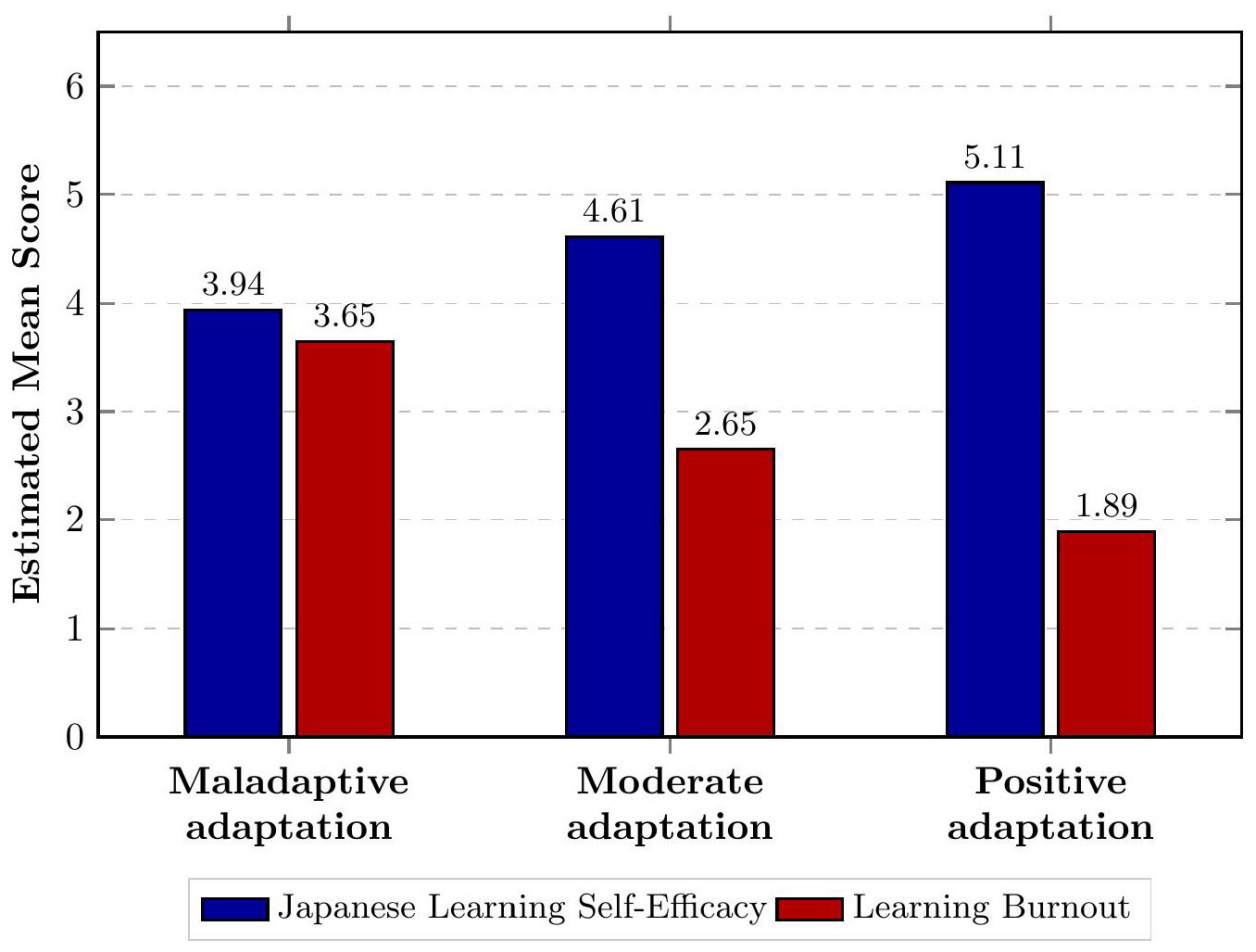
Estimated means of Japanese learning self-efficacy and learning burnout across the time 3 latent profiles.

To examine the practical significance of the identified adaptation profiles, Japanese learning self-efficacy and learning burnout at Time 3 were compared across the three latent profiles. The results are presented in Table 8, and the corresponding mean differences are illustrated in Figure 2. Significant profile differences were found for both distal outcomes. For Japanese learning self-efficacy, the omnibus test was significant, *F* = 42.13, *p* < 0.001. Learners in the positive adaptation profile reported the highest level of self-efficacy (*M* = 5.11), followed by those in the moderate adaptation profile (*M* = 4.61), whereas those in the maladaptive adaptation profile reported the lowest level (*M* = 3.94). Pairwise comparisons further indicated that all three profiles differed significantly from one another, with self-efficacy increasing progressively from maladaptive adaptation to moderate adaptation and then to positive adaptation. A similarly significant pattern emerged for learning burnout, *F* = 100.69, *p* < 0.001. Learners in the maladaptive adaptation profile showed the highest level of burnout (*M* = 3.65), followed by those in the moderate adaptation profile (*M* = 2.65), whereas learners in the positive adaptation profile reported the lowest level of burnout (*M* = 1.89). Pairwise comparisons again showed significant differences between all three profiles, indicating a clear gradient in which more adaptive profile membership was associated with lower levels of burnout. These findings support the practical relevance of the identified latent profiles. Learners classified into the positive adaptation profile not only exhibited lower technostress and higher resilience, but also reported more favorable learning-related outcomes, whereas those in the maladaptive adaptation profile showed the least favorable pattern.

**Table 8:** Distal outcomes by time 3 profile membership.

Outcome	Maladaptive adaptation	Moderate adaptation	Positive adaptation	Wald test	*p*	Pairwise comparison
Japanese learning self-efficacy	3.94	4.61	5.11	χ^2^*Wald*^(2)^= 42.13	< 0.001	Moderate > Maladaptive; Positive > Maladaptive; Positive > Moderate
Learning burnout	3.65	2.65	1.89	χ^2^*Wald*^(2)^= 100.69	< 0.001	Moderate < Maladaptive; Positive < Maladaptive; Positive < Moderate

Profile-specific values are BCH-adjusted means.

Omnibus differences are Wald chi-square tests with two degrees of freedom, and classification uncertainty is accounted for through BCH weights.

### 4.6 Auxiliary analyses: role of the control variables

Auxiliary analyses were conducted to determine whether the seven background variables predicted Time 1 profile membership and whether the Time 3 distal-outcome differences remained after adjustment for these characteristics. As shown in Table 9, the R3STEP multinomial logistic regression indicated that several learner characteristics significantly predicted initial profile membership. Relative to the maladaptive adaptation profile, learners with higher self-rated Japanese proficiency (*OR* = 1.68, *p* = 0.003), more frequent AI use (*OR* = 1.42, *p* = 0.012), and prior AI-related training (*OR* = 2.16, *p* < 0.001) had significantly greater odds of membership in the positive adaptation profile. Prior AI-related training also increased the odds of membership in the moderate adaptation profile relative to the maladaptive adaptation profile (*OR* = 1.58, *p* = 0.024). Gender, age, year of study, and major status were not statistically significant predictors of initial profile membership (all *p*>0.10). The background variables were not specified as predictors of the Time 1–Time 2 or Time 2–Time 3 transition probabilities. Accordingly, the transition probabilities reported in the main analyses were obtained from the unconditional latent transition model. Finally, manual BCH-weighted regression models were estimated to determine whether the profile differences in the distal outcomes remained after adjustment for all seven background variables. The adjusted profile differences remained statistically significant for Japanese learning self-efficacy, $\chi_{Wald}^{2}\left(2\right)=38.85$, *p* < 0.001, and learning burnout, $\chi_{Wald}^{2}\left(2\right)=92.41$, *p* < 0.001. These findings indicate that the observed profile differences in the distal outcomes were not fully attributable to the seven measured demographic and background characteristics.

**Table 9:** Auxiliary multinomial logistic regression predicting Time 1 profile membership.

2–4(lr)5–7 Predictor	Moderate vs. Maladaptive			Positive vs. Maladaptive		
	**B (SE)**	**OR**	***p***	**B (SE)**	**OR**	***p***
Gender (1 = Female)	−0.12 (0.21)	0.89	0.568	0.05 (0.24)	1.05	0.835
Age	0.03 (0.08)	1.03	0.708	−0.04 (0.09)	0.96	0.658
Year of study	0.08 (0.11)	1.08	0.462	0.14 (0.12)	1.15	0.242
Major status (1 = Japanese major)	0.15 (0.20)	1.16	0.453	0.22 (0.22)	1.25	0.318
Self-rated Japanese proficiency	0.21 (0.13)	1.23	0.106	0.52 (0.17)	1.68	0.003
Frequency of AI use	0.16 (0.11)	1.17	0.145	0.35 (0.14)	1.42	0.012
Prior AI training (1 = Yes)	0.46 (0.20)	1.58	0.024	0.77 (0.22)	2.16	< 0.001

The maladaptive adaptation profile was the reference category.

B, unstandardized multinomial logit coefficient; SE, standard error; OR, odds ratio (eB).

All seven predictors were entered simultaneously.

## 5 Discussion

### 5.1 Summary of the main findings

The present study examined psychological adaptation to AI-assisted Japanese language learning from a person-centered and longitudinal perspective. Four main findings emerged. First, learners could be meaningfully classified into three distinct profiles based on technostress and resilience, namely maladaptive adaptation, moderate adaptation, and positive adaptation. Second, the same profile structure was observed across the three waves, indicating that the underlying pattern of psychological adaptation was conceptually stable over time. Third, profile membership was not fixed. Although a substantial proportion of learners remained in the same profile across measurement occasions, meaningful transitions were also observed, with the overall distribution showing a gradual shift away from maladaptive adaptation and toward more positive adaptation across the semester. Fourth, the identified profiles were practically meaningful, as learners in more adaptive profiles reported higher Japanese learning self-efficacy and lower learning burnout than those in less adaptive profiles. Taken together, these findings suggest that adaptation to AI-assisted Japanese language learning is best understood as a heterogeneous and dynamic psychological process rather than as a single, static continuum (Ferguson et al., 2020; Nylund-Gibson et al., 2023; Ryoo et al., 2018).

### 5.2 Interpreting the latent profiles of psychological adaptation

One important contribution of the present study lies in showing that learners did not respond to AI-assisted Japanese language learning in a uniform manner. Under Job Demands–Resources theory, the three retained profiles can be interpreted as distinct demand–resource regimes rather than as arbitrary statistical groupings (Bakker and Demerouti, 2017; Demerouti et al., 2001; Jagodics and Szabó, 2023). The maladaptive adaptation profile represents a demand-dominant condition consistent with the health-impairment pathway: elevated technostress consumes cognitive and emotional energy, while limited resilience constrains coping and recovery. The positive adaptation profile represents a resource-advantaged condition consistent with a resource-supported motivational pathway, in which lower technological demands and stronger resilience facilitate sustained coping and engagement. The moderate adaptation profile represents an intermediate condition in which neither demands nor resources clearly predominate, leaving learners functional but more psychologically contingent and potentially open to movement in either direction. This interpretation explains why the profiles are psychologically distinct even though they can also be ordered from less adaptive to more adaptive. They represent different relations between experienced demands and available resources: resource insufficiency, provisional balance, and resource-supported adaptation. The profile solution therefore extends prior variable-centered work on AI-assisted and technology-enhanced learning by demonstrating that similar technological environments may correspond to different adaptive regimes across learners (Jiang, 2022; Wang et al., 2021; Xu and Wang, 2024). The maladaptive adaptation profile was marked by relatively high technostress and low resilience. Psychologically, this pattern suggests that some learners may encounter AI-assisted learning primarily as a source of cognitive burden, uncertainty, and reduced coping capacity. This interpretation is consistent with the broader technostress literature, which has long argued that digital tools may undermine functioning when they are experienced as overly complex, intrusive, or difficult to manage (Kumar, 2024; Ragu-Nathan et al., 2008; Tarafdar et al., 2007). In the context of AI-assisted language learning, these pressures may be especially salient because learners are not only using technology, but also monitoring the reliability of AI-generated content, deciding when to trust or question feedback, and regulating their own use of intelligent tools (Barrett and Pack, 2023; Li et al., 2024; Teng, 2024).

The positive adaptation profile showed the opposite pattern, namely relatively low technostress and high resilience. This combination suggests that some learners were able to engage with AI-assisted Japanese learning in a comparatively confident and well-regulated manner. Rather than perceiving AI as a destabilizing force, they may have incorporated it into their study routines in a way that preserved psychological balance and adaptive functioning. This interpretation is consistent with research showing that resilience reflects not simply the absence of strain, but a positive capacity to respond effectively to challenge and recover from setbacks (Campbell-Sills and Stein, 2007; Connor and Davidson, 2003; Hartley, 2011). The present findings therefore support the view that resilience can function as an important personal resource in digitally demanding learning environments. The moderate adaptation profile was the largest group at all three waves. This is theoretically meaningful. Rather than indicating a weak or uninteresting pattern, the moderate profile may represent a normative and transitional form of adaptation in which learners are neither strongly distressed nor fully comfortable in AI-assisted learning. Such learners may be managing the demands of AI reasonably well, yet still remain psychologically malleable. In this sense, the moderate profile may capture the most common state of adjustment in the early and middle stages of engagement with AI-assisted Japanese language learning.

In latent profile analysis of Chinese university students using large language models for EFL learning, Wang et al. (2025b) identified enthusiastic explorers, adaptable learners, and ambitious-anxious pioneers based on engagement, self-efficacy, and anxiety. The presence of contrasting psychological configurations in that study supports the broader premise that students do not engage with AI-assisted language learning uniformly.

### 5.3 Stability and transitions of adaptation profiles over time

Another important contribution of the study lies in its demonstration that psychological adaptation was dynamic rather than static. Although the three profiles retained a stable conceptual structure across the semester, learners did not remain perfectly fixed within them. This finding is consistent with the logic of latent transition analysis, which emphasizes that latent statuses may show both continuity and movement over time (Nylund-Gibson et al., 2023; Ryoo et al., 2018). In the present study, the overall pattern of change was not random. Instead, it suggested a gradual tendency toward improved adaptation. The positive adaptation profile displayed the highest temporal stability. This suggests that once learners reached a pattern characterized by low technostress and high resilience, they were relatively likely to maintain that psychological advantage across subsequent waves. One plausible interpretation is that learners in this profile may have already developed more effective ways of integrating AI tools into their language learning routines. As recent studies on generative AI in language learning suggest, learners who use AI in a more purposeful and self-directed way may be better able to benefit from its flexibility without becoming overwhelmed by its demands (Li et al., 2025, 2024; Teng, 2024).

By contrast, the maladaptive adaptation profile showed lower stability and a stronger tendency to move into the moderate adaptation profile than directly into the positive adaptation profile. This pattern is psychologically plausible. A shift from high strain and low resilience to a more positive state is unlikely to occur abruptly. It is more realistic that learners first move into an intermediate position in which technostress becomes more manageable and resilience begins to recover, before eventually reaching a more adaptive configuration. In other words, improvement in psychological adaptation appears to be incremental rather than immediate. The moderate adaptation profile was particularly informative in this regard. It was both the largest and one of the most transitional groups, serving as a midpoint from which learners could move either toward more adaptive or less adaptive functioning. This suggests that the moderate profile may be the most practically important group for intervention. Learners in this profile do not appear severely maladjusted, but neither are they fully protected. Their position may be especially sensitive to contextual supports, self-regulatory habits, and the quality of their experiences with AI-assisted learning.

At the aggregate level, the decreasing proportion of learners in the maladaptive adaptation profile and the increasing proportion in the positive adaptation profile suggest that many learners became better adapted to AI-assisted Japanese language learning over the semester. Greater familiarity with AI remains one plausible explanation, but a demand–resource perspective suggests that this change may also reflect the gradual development of learner resources. One such resource is AI literacy, which includes the capacity to understand, critically evaluate, and use AI technologies appropriately (Long and Magerko, 2020). As learners gain experience in formulating prompts, checking the accuracy of generated responses, recognizing the limitations of AI tools, and deciding when external verification is necessary, the complexity and uncertainty associated with AI use may decline. These developing competencies could reduce technostress even when the technological environment itself remains unchanged. The development of self-regulated learning and the integration of AI into stable learning routines offer complementary explanations. Self-regulated learners actively set goals, monitor their performance, evaluate the effectiveness of their strategies, and modify their behavior when necessary (Zimmerman, 2002). Across the semester, learners may have become more selective about when and how to use AI, for example, by distinguishing between tasks for which AI feedback was useful and tasks that required independent judgment or teacher guidance. Repeated and increasingly purposeful use may also have embedded particular AI tools into regular Japanese learning routines, reducing the decision-making burden associated with tool selection and enabling successful experiences to strengthen confidence and resilience (Li et al., 2024; Teng, 2024). This interpretation is consistent with the predominantly stepwise transition pattern, in which learners in the maladaptive adaptation profile were more likely to move first into the moderate adaptation profile than directly into the positive adaptation profile. These explanations should nevertheless be interpreted cautiously. AI literacy, self-regulated learning, and routine integration were not directly assessed in the present study, and the observed transitions therefore do not establish that changes in these factors caused the increase in positive adaptation. Rather, they represent theoretically plausible mechanisms that should be examined directly in future longitudinal research. Moreover, the persistence of a maladaptive adaptation group across all three waves indicates that increased exposure to AI does not automatically produce positive adaptation. Some learners may continue to experience a mismatch between technological demands and their available coping resources and may therefore require more structured instructional and psychological support.

### 5.4 Distal outcomes and the practical significance of the profiles

The comparison of distal outcomes further confirmed that the identified profiles were not merely statistical artifacts. Learners in the positive adaptation profile reported the highest level of Japanese learning self-efficacy and the lowest level of learning burnout, whereas those in the maladaptive adaptation profile showed the least favorable pattern. These differences provide important external evidence for the substantive meaning of the latent profiles. The self-efficacy findings are especially notable. Self-efficacy is a domain-specific belief about one's capability to perform required tasks successfully, and prior research has shown that it is closely linked to persistence, confidence, and adaptive engagement in language learning (Fryer et al., 2020; Wang et al., 2014). In the present study, learners with lower technostress and higher resilience also reported stronger confidence in their ability to learn Japanese effectively. This finding suggests that adaptive psychological functioning in an AI-assisted environment is not limited to feeling less strained. It also includes feeling more capable of handling language learning demands.

The burnout findings point in the same direction. Burnout among students is typically characterized by exhaustion, cynicism, and reduced academic efficacy, and it has increasingly been interpreted through a demand-resource framework (Hu and Schaufeli, 2009; Jagodics and Szabó, 2023; Schaufeli et al., 2002). From this perspective, technostress may operate as a learning demand, whereas resilience may function as a personal resource. The present results are consistent with that interpretation. Learners in the maladaptive adaptation profile, who combined higher technostress with lower resilience, reported the highest burnout. Those in the positive adaptation profile reported the lowest burnout. This pattern strengthens the claim that psychological adaptation to AI-assisted language learning has direct implications for learners' broader academic functioning. The distal outcome results show that the latent profiles identified in this study are educationally meaningful. They do not simply distinguish learners on the basis of internal psychological indicators. Rather, they are linked to differences in confidence and strain that are central to learners' ongoing success in Japanese language learning.

### 5.5 Theoretical and practical implications

The present study has several theoretical implications. First, it contributes to the growing literature on AI-assisted language learning by reframing this context as a setting of psychological adaptation rather than merely a site of technological adoption or instructional effectiveness (Jiang, 2022; Li et al., 2025; Xu and Wang, 2024). This shift in emphasis is important because it highlights that learners' experiences with AI are shaped not only by what the technology can do, but also by how learners psychologically manage its demands. Second, the findings extend the technostress literature into a more specific and emerging language-learning context. Prior work has shown that technostress can undermine wellbeing and performance in both organizational and educational settings (Kumar, 2024; Ragu-Nathan et al., 2008; Tarafdar et al., 2007; Wang et al., 2021). The present study suggests that its role in AI-assisted Japanese learning cannot be fully understood in isolation. Instead, technostress should be considered jointly with resilience, because the same level of technological demand may have different implications depending on the learner's adaptive resources. Third, the study supports the value of a person-centered and longitudinal perspective in educational psychology. By identifying latent profiles and their transitions over time, the study moves beyond average associations and demonstrates that adaptation unfolds differently across subgroups of learners (Ferguson et al., 2020; Nylund-Gibson et al., 2023; Ryoo et al., 2018). This perspective offers a more nuanced understanding of how learners respond to AI-assisted study and why some appear to benefit more readily than others. The study also has practical implications. For instructors and curriculum designers, the results suggest that support for AI-assisted Japanese learning should not focus exclusively on technical skills. It should also address learners' psychological adjustment. Training that helps students evaluate AI output critically, use AI strategically, and set appropriate boundaries may reduce unnecessary technostress and make the learning environment more manageable (Barrett and Pack, 2023; Li et al., 2024; Teng, 2024).

The results suggest that learners may benefit from differentiated forms of support. This interpretation is consistent with the Q-method findings of Wang et al. (2025a), who identified high-structure, comfort-seeking, and practice-driven learners with different preferences for pedagogical scaffolding, emotional reassurance, technical assistance, autonomy support, and feedback in AI-assisted EFL learning. In the present context, learners in the maladaptive adaptation profile may benefit from clearer guidance and reassurance, whereas those in the moderate adaptation profile may respond to support for self-regulation and confidence building. Learners in the positive adaptation profile may be ready for more autonomous and reflective uses of AI. More broadly, the findings suggest that educational practice should treat successful AI integration not only as a matter of access and usage, but also as a matter of psychological sustainability. A learning environment that is technologically rich but psychologically overwhelming is unlikely to promote durable language development. By contrast, environments that reduce avoidable strain while strengthening adaptive resources may be better positioned to support both effective learning and student wellbeing.

### 5.6 Limitations and future directions

Several limitations should be acknowledged. First, the study relied on self-report measures, which may be influenced by common method variance and subjective response tendencies. Although the longitudinal design and person-centered analysis reduce some concerns, future research would benefit from incorporating additional data sources, such as behavioral indicators of AI use or teacher evaluations of learning engagement. Second, the sample was drawn from learners of Japanese in a specific higher education context. The generalizability of the profile structure to other languages, institutions, or cultural settings remains to be established. Third, the study covered one academic semester. Although this design made it possible to detect meaningful transitions, longer-term follow-up would be useful for examining whether adaptive improvements are sustained over time.

The explanatory interpretations offered in the Discussion also require caution. AI literacy, self-regulated learning, routine integration, and perceived teacher support were not directly measured; consequently, they should be regarded as theoretically plausible explanations for the observed profile patterns rather than as empirically tested mechanisms. Although the longitudinal design establishes temporal patterns of stability and change, it does not demonstrate that these factors caused learners to move between profiles. Moreover, evidence from AI-assisted EFL learning provides relevant contextual support but does not directly validate the present findings because the target language, profile indicators, and analytical purposes differ.

Future studies could extend the present work in several ways. One direction would be to incorporate antecedent variables such as AI literacy, perceived instructional support, or self-regulated learning strategies in order to explain why learners enter or leave particular profiles. Another would be to examine additional outcomes, such as learning engagement, achievement, persistence, or observable patterns of AI use. It would also be valuable to compare profile structures across different target languages or across different types of AI-supported learning tasks. Such work would deepen our understanding of how psychological adaptation develops and how it can be supported more effectively in AI-mediated language education.

## 6 Conclusion

This study examined psychological adaptation to AI-assisted Japanese language learning from a person-centered and longitudinal perspective. By focusing on technostress and resilience, the study showed that learners did not respond to AI-assisted learning in a uniform way, but instead formed three distinct profiles of psychological adaptation: maladaptive adaptation, moderate adaptation, and positive adaptation. These profiles were not only conceptually stable across the semester, but also dynamically changeable, with the overall pattern indicating a gradual movement toward more adaptive functioning over time. The findings therefore underscore that AI-assisted language learning is not simply a matter of technological access or instructional efficiency. It is also a psychological process involving stress regulation, coping capacity, confidence, and vulnerability to burnout.

The results further indicated that the adaptation profiles were associated with Japanese learning self-efficacy and learning burnout within the present sample. Learners characterized by lower technostress and higher resilience reported more favorable motivational and emotional outcomes, whereas those characterized by higher technostress and lower resilience reported the least favorable pattern. Because these relationships were based on observational self-report data, they should not be interpreted as evidence that profile membership caused the distal outcomes. Rather, the findings suggest that psychological adaptation may be educationally relevant alongside technological access and instructional design. Attending to how learners experience and manage AI-supported study may therefore help educators develop more psychologically sustainable approaches to language learning, although the effectiveness of specific support strategies requires direct evaluation in future research.

## Data Availability

The original contributions presented in the study are included in the article/supplementary material, further inquiries can be directed to the corresponding authors.

## References

[B1] Alshumaimeri Y. A. Alshememry A. K. (2024). The extent of AI applications in EFL learning and teaching. IEEE Trans. Learn. Technol. 17, 653–663. doi: 10.1109/TLT.2023.3322128

[B2] Asparouhov T. Muthén B. O. (2014). Auxiliary variables in mixture modeling: three-step approaches using Mplus. Struct. Equat. Model. A Multidiscipl. J. 21, 329–341. doi: 10.1080/10705511.2014.915181

[B3] Bakker A. B. Demerouti E. (2017). Job demands–resources theory: taking stock and looking forward. J. Occup. Health Psychol. 22, 273–285. doi: 10.1037/ocp0000056 27732008

[B4] Bandura A. (1997). Self-Efficacy: The Exercise of Control. New York, NY: W. H. Freeman.

[B5] Barrett A. Pack A. (2023). Not quite eye to A.I.: student and teacher perspectives on the use of generative artificial intelligence in the writing process. Int. J. Educ. Technol. High. Educ. 20:59. doi: 10.1186/s41239-023-00427-0

[B6] Bolck A. Croon M. Hagenaars J. (2004). Estimating latent structure models with categorical variables: one-step versus three-step estimators. Polit. Anal. 12, 3–27. doi: 10.1093/pan/mph001

[B7] Campbell-Sills L. Stein M. B. (2007). Psychometric analysis and refinement of the Connor–Davidson Resilience Scale (CD-RISC): validation of a 10-item measure of resilience. J. Traum. Stress 20, 1019–1028. doi: 10.1002/jts.20271 18157881

[B8] Cazan A.-M. David L. T. Trută C. Maican C. I. Henter R. Năstasă L. E. . (2024). Technostress and time spent online. a cross-cultural comparison for teachers and students. Front. Psychol. 15:1377200. doi: 10.3389/fpsyg.2024.1377200 39049944 PMC11267416

[B9] Chen F. F. (2007). Sensitivity of goodness of fit indexes to lack of measurement invariance. Struct. Equat. Model. A Multidiscipl. J. 14, 464–504. doi: 10.1080/10705510701301834

[B10] Connor K. M. Davidson J. R. T. (2003). Development of a new resilience scale: the Connor–Davidson Resilience Scale (CD-RISC). Depress. Anxiety 18, 76–82. doi: 10.1002/da.10113 12964174

[B11] Crompton H. Burke D. (2023). Artificial intelligence in higher education: the state of the field. Int. J. Educ. Technol. High. Educ. 20:22. doi: 10.1186/s41239-023-00392-8

[B12] Demerouti E. Bakker A. B. Nachreiner F. Schaufeli W. B. (2001). The job demands–resources model of burnout. J. Appl. Psychol. 86, 499–512. doi: 10.1037/0021-9010.86.3.499 11419809

[B13] Enders C. K. Bandalos D. L. (2001). The relative performance of full information maximum likelihood estimation for missing data in structural equation models. Struct. Equat. Model. A Multidiscipl. J. 8, 430–457. doi: 10.1207/S15328007SEM0803_5

[B14] Etherton K. Steele-Johnson D. Salvano K. Kovacs N. (2022). Resilience effects on student performance and well-being: the role of self-efficacy, self-set goals, and anxiety. J. Gener. Psychol. 149, 279–298. doi: 10.1080/00221309.2020.1835800 33111653

[B15] Ferguson S. L. Moore E. W. G. Hull D. M. (2020). Finding latent groups in observed data: a primer on latent profile analysis in Mplus for applied researchers. Int. J. Behav. Dev. 44, 458–468. doi: 10.1177/0165025419881721

[B16] Fryer L. K. Thompson A. Nakao K. Howarth M. Gallacher A. (2020). Supporting self-efficacy beliefs and interest as educational inputs and outcomes: framing AI and human partnered task experiences. Learn. Indiv. Differ. 80:101850. doi: 10.1016/j.lindif.2020.101850

[B17] Gong Z. Wang H. Zhong M. Shao Y. (2023). College students' learning stress, psychological resilience and learning burnout: status quo and coping strategies. BMC Psych. 23:389. doi: 10.1186/s12888-023-04783-z 37268888 PMC10236398

[B18] Guan L. Li S. Gu M. M. (2024). AI in informal digital english learning: a meta-analysis of its effectiveness on proficiency, motivation, and self-regulation. Comput. Educ. Artif. Intell. 7:100323. doi: 10.1016/j.caeai.2024.100323

[B19] Hallquist M. N. Wiley J. F. (2018). MplusAutomation: an R package for facilitating large-scale latent variable analyses in Mplus. Struct. Equat. Model. A Multidiscipl. J. 25, 621–638. doi: 10.1080/10705511.2017.1402334 30083048 PMC6075832

[B20] Hartley M. T. (2011). Examining the relationships between resilience, mental health, and academic persistence in undergraduate college students. J. Amer. Coll. Health 59, 596–604. doi: 10.1080/07448481.2010.515632 21823954

[B21] Hu L. Bentler P. M. (1999). Cutoff criteria for fit indexes in covariance structure analysis: Conventional criteria versus new alternatives. Struct. Equat. Model. A Multidiscipl. J. 6, 1–55. doi: 10.1080/10705519909540118

[B22] Hu Q. Schaufeli W. B. (2009). The factorial validity of the Maslach Burnout Inventory–Student Survey in China. Psycholog. Rep. 105, 394–408. doi: 10.2466/PR0.105.2.394-408 19928601

[B23] Jagodics B. Szabó É. (2023). Student burnout in higher education: a demand-resource model approach. Trends Psychol. 31, 757–776. doi: 10.1007/s43076-021-00137-4

[B24] Jiang R. (2022). How does artificial intelligence empower EFL teaching and learning nowadays? a review on artificial intelligence in the EFL context. Front. Psychol. 13:1049401. doi: 10.3389/fpsyg.2022.1049401 36467167 PMC9709327

[B25] Kumar P. S. (2024). Technostress: a comprehensive literature review on dimensions, impacts, and management strategies. Comput. Hum. Behav. Rep. 16:100475. doi: 10.1016/j.chbr.2024.100475

[B26] Lee H. Lee J. H. (2022). The effects of robot-assisted language learning: a meta-analysis. Educ. Res. Rev. 35:100425. doi: 10.1016/j.edurev.2021.100425

[B27] Li B. Tan Y. L. Wang C. Lowell V. (2025). Two years of innovation: a systematic review of empirical generative AI research in language learning and teaching. Comput. Educ. Artif. Intell. 9:100445. doi: 10.1016/j.caeai.2025.100445

[B28] Li Z. Wang C. Bonk C. J. (2024). Exploring the utility of ChatGPT for self-directed online language learning. Online Learn. 28, 157–180. doi: 10.24059/olj.v28i3.4497

[B29] Liu W. Zhang R. Wang H. Rule A. Wang M. Abbey C. . (2024). Association between anxiety, depression symptoms, and academic burnout among chinese students: the mediating role of resilience and self-efficacy. BMC Psychol. 12:335. doi: 10.1186/s40359-024-01823-5 38849921 PMC11162059

[B30] Long D. Magerko B. (2020). “What is AI literacy? competencies and design considerations,” in Proceedings of the 2020 CHI Conference on Human Factors in Computing Systems, (New York, NY: Association for Computing Machinery), 1–16. doi: 10.1145/3313831.3376727

[B31] Nylund K. L. Asparouhov T. Muthén B. O. (2007). Deciding on the number of classes in latent class analysis and growth mixture modeling: a Monte Carlo simulation study. Struct. Equat. Model. A Multidiscipl. J. 14, 535–569. doi: 10.1080/10705510701575396

[B32] Nylund-Gibson K. Garber A. C. Carter D. B. Chan M. Arch D. A. N. Simon O. . (2023). Ten frequently asked questions about latent transition analysis. Psychol. Methods 28, 284–300. doi: 10.1037/met0000486 35834194

[B33] Qiao H. Zhao A. (2023). Artificial intelligence-based language learning: illuminating the impact on speaking skills and self-regulation in chinese EFL context. Front. Psychol. 14:1255594. doi: 10.3389/fpsyg.2023.1255594 38022973 PMC10652775

[B34] Ragu-Nathan T. S. Tarafdar M. Ragu-Nathan B. S. Tu Q. (2008). The consequences of technostress for end users in organizations: conceptual development and empirical validation. Inf. Syst. Res. 19, 417–433. doi: 10.1287/isre.1070.0165

[B35] Rosseel Y. (2012). lavaan: an R package for structural equation modeling. J. Statist. Softw. 48, 1–36. doi: 10.18637/jss.v048.i02

[B36] Ryoo J. H. Wang C. Swearer S. M. Hull M. Shi D. (2018). Longitudinal model building using latent transition analysis: an example using school bullying data. Front. Psychol. 9:675. doi: 10.3389/fpsyg.2018.00675 29867652 PMC5953336

[B37] Saleem F. Chikhaoui E. Malik M. I. (2024). Technostress in students and quality of online learning: role of instructor and university support. Front. Educ. 9:1309642. doi: 10.3389/feduc.2024.1309642

[B38] Schaufeli W. B. Martínez I. M. Marques Pinto A. Salanova M. Bakker A. B. (2002). Burnout and engagement in university students: a cross-national study. J. Cross-Cult. Psychol. 33, 464–481. doi: 10.1177/0022022102033005003

[B39] Sousa V. D. Rojjanasrirat W. (2011). Translation, adaptation and validation of instruments or scales for use in cross-cultural health care research: a clear and user-friendly guideline. J. Eval. Clin. Pract. 17, 268–274. doi: 10.1111/j.1365-2753.2010.01434.x 20874835

[B40] Tarafdar M. Tu Q. Ragu-Nathan B. S. Ragu-Nathan T. S. (2007). The impact of technostress on role stress and productivity. J. Manag. Inf. Syst. 24, 301–328. doi: 10.2753/MIS0742-1222240109

[B41] Teng M. F. (2024). “ChatGPT is the companion, not enemies”: EFL learners' perceptions and experiences in using ChatGPT for feedback in writing. Comput. Educ. Artif. Intell. 7:100270. doi: 10.1016/j.caeai.2024.100270

[B42] Vandenberg R. J. Lance C. E. (2000). A review and synthesis of the measurement invariance literature: suggestions, practices, and recommendations for organizational research. Org. Res. Methods 3, 4–70. doi: 10.1177/109442810031002

[B43] Wang C. Kim D.-H. Bai R. Hu J. (2014). Psychometric properties of a self-efficacy scale for english language learners in China. System 44, 24–33. doi: 10.1016/j.system.2014.01.015

[B44] Wang L. Shi Z. Zhang Y. Zhang Z. (2010). Psychometric properties of the 10-item Connor–Davidson Resilience Scale in chinese earthquake victims. Psych. Clin. Neurosci. 64, 499–504. doi: 10.1111/j.1440-1819.2010.02130.x 20923429

[B45] Wang Q. Gao Y. Sun J. (2025a). What matters most? AQ-method study on Chinese university students' perceived teacher support in AI-assisted EFL learning. Eur. J. Educ. 60:e70327. doi: 10.1111/ejed.70327

[B46] Wang Q. Gao Y. Wang X. (2025b). Exploring engagement, self-efficacy, and anxiety in large language model EFL learning: a latent profile analysis of chinese university students. Int. J. Hum.–Comput. Interact. 41, 7815–7824. doi: 10.1080/10447318.2024.2400403

[B47] Wang X. Li Z. Ouyang Z. Xu Y. (2021). The achilles heel of technology: how does technostress affect university students' wellbeing and technology-enhanced learning? Int. J. Environ. Res. Public Health 18:12322. doi: 10.3390/ijerph182312322 34886048 PMC8656752

[B48] Wei L. (2023). Artificial intelligence in language instruction: impact on english learning achievement, L2 motivation, and self-regulated learning. Front. Psychol. 14:1261955. doi: 10.3389/fpsyg.2023.1261955 38023040 PMC10658009

[B49] Xu T. Wang H. (2024). The effectiveness of artificial intelligence on english language learning achievement. System 125:103428. doi: 10.1016/j.system.2024.103428

[B50] Zhai C. Wibowo S. (2023). A systematic review on artificial intelligence dialogue systems for enhancing english as a foreign language students' interactional competence in the university. Comput. Educ. Artif. Intell. 4:100134. doi: 10.1016/j.caeai.2023.100134

[B51] Zhai X. Li S. (2025). The roles of growth mindset, resilience, and self-efficacy in student engagement with AI-enhanced chinese learning: a self-determination theory perspective. Learn. Motiv. 92:102183. doi: 10.1016/j.lmot.2025.102183

[B52] Zimmerman B. J. (2002). Becoming a self-regulated learner: an overview. Theory Pract. 41, 64–70. doi: 10.1207/s15430421tip4102_2

